# Fistule esotrachéale post-traumatique causée par un couteau: un rapport de cas

**DOI:** 10.11604/pamj.2024.48.102.41715

**Published:** 2024-07-12

**Authors:** Moussa Seck Diop, Souleymane Diatta, Fabrice Arroye, Temadjibaye Danalta, Marie Amy Kébé, Marème Soda Mbaye, Mory Camara, Kondo Bignandi, Dialtabé Ba, Amadou Gabriel Ciss, Assane Ndiaye

**Affiliations:** 1 Service de Chirurgie Thoracique et Cardiovasculaire, Centre Hospitalier National Universitaire de Fann, Dakar, Sénégal

**Keywords:** Esotracheal fistula, stabbing wound, Senegal, case report, Fistule œso-trachéale, arme blanche, Sénégal, cas clinique

## Abstract

les fistules oeso-trachéales (FOT) secondaires à une plaie par arme blanche sont rares et sous-diagnostiquées. Le scanner thoracique et l´opacification œsophagienne aident au diagnostic. Leur traitement est chirurgical. Nous rapportons le cas d´un patient ayant présenté une FOT après avoir reçu un couteau dans le dos. Admis à 6 heures, il se plaignait d´une douleur médio-thoracique. L´examen retrouvait une plaie paravertébrale droite, avec la lame du couteau en intrathoracique. L´imagerie montrait la lame traversant le médiastin supérieur, un hémo-pneumothorax droit et des bulles d´air médiastinales. L´exploration chirurgicale retrouvait une petite FOT réparée par suture directe des orifices trachéal et œsophagien avec plastie de recouvrement. L´alimentation fut autorisée à J14 après un contrôle n´objectivant pas de fistule résiduelle. Les suites opératoires furent simples. Ainsi, les FOT sont rares mais potentiellement graves. Leur diagnostic précoce et leur traitement chirurgical encadrés d´une bonne nutrition et une gestion de l´infection permettent de réduire leur morbi-mortalité.

## Abstract (en)

Traumatic esophageal-tracheal fistulas (ETFs) secondary to a stab wound are rare and often underdiagnosed. Chest computed tomography (CT) scan and opacification of the esophageal lumen help in diagnosis. Surgery is required for treatment. We here report the case of a patient who developed an ETF after being stabbed in the back. Admitted six hours after the incident, he complained of mid-thoracic pain. Physical examination revealed a right paravertebral wound with intrathoracic knife blade. Imagery showed the blade traversing the upper mediastinum, right hemopneumothorax, and mediastinal air bubbles. Surgical exploration revealed a small ETF which was repaired by direct suture of the tracheal and oesophageal orifices with a covering flap. Oral feeding was allowed at D14 after a check-up revealed no residual fistula. The postoperative course was uncomplicated. Esophageal-tracheal fistulas are rare and potentially serious. Early diagnosis and surgical management, combined with good nutrition and infection management, help reduce their morbidity and mortality.

## Introduction

Les fistules oeso-trachéales se définissent par une communication anormale entre l´œsophage et l´arbre trachéo-bronchique. Elles peuvent se faire à travers un défect congénital ou acquis. Les FOT post-traumatiques sont extrêmement rares, encore plus celles secondaires à une plaie par arme blanche [1]. La non spécificité des signes cliniques conduit souvent à un diagnostic tardif, source de complications. Nous rapportons le cas exceptionnel d´un patient qui a présenté une fistule oeso-trachéale post-traumatique, avec une arme blanche restée en intra-thoracique, pris en charge chirurgicalement dans notre service.

## Patient et observation

**Informations sur le patient:** il s´agissait d´un patient de 17 ans, mécanicien de profession, sans antécédent pathologique particulier, qui aurait reçu un coup de poignard dans le dos au décours d´une rixe. Il aurait présenté immédiatement une douleur médio-thoracique vive sans dyspnée ni toux ni hémoptysie et un saignement de faible abondance au niveau de la plaie. Il fut transporté dans notre structure en urgence.

**Figure 1:**
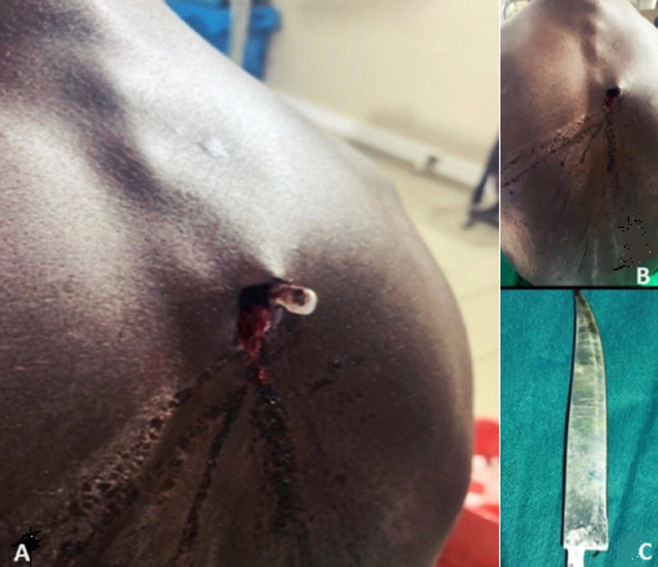
lame du couteau dans le thorax; A, B): bout du couteau faisant saillie au niveau du dos, à droite du rachis en regard de D5; C) couteau retiré mesurant 15x4 cm

**Résultats cliniques:** à l´admission, à six heures du traumatisme, le patient était stable sur le plan neurologique, hémodynamique et respiratoire. Les muqueuses conjonctivales étaient colorées. Localement, l´examen avait objectivé une plaie thoracique postérieure linéaire verticale d´environ 3 cm de long, en paravertébral droit à hauteur de D4-D5, non soufflante, sans saignement actif, avec visualisation du bout de la lame du couteau en intra-thoracique (Figure 1 A, B, C). Les champs pulmonaires étaient libres. Les bruits du cœur étaient bien perçus, réguliers, sans bruit surajouté, non déviés. Les pouls étaient bien perçus et symétriques à tous les sites. Il n´y avait pas de déficit sensitif ou moteur aux membres supérieurs et inférieurs.

**Figure 2:**
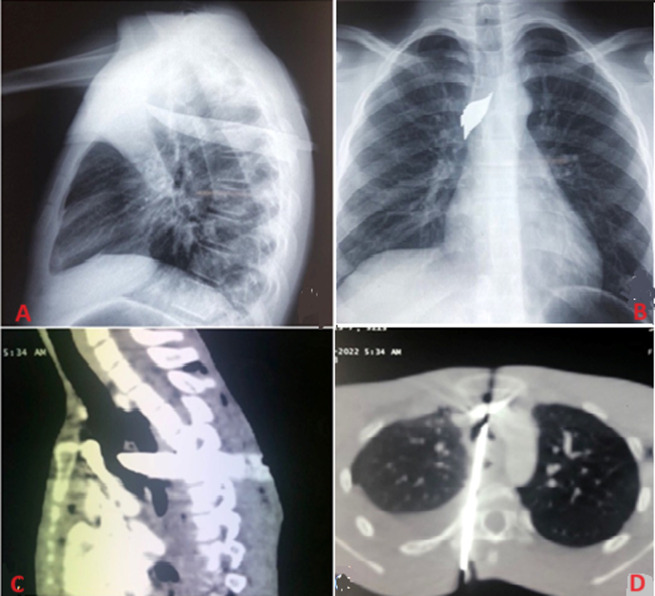
imagerie montrant le couteau dans le thorax; A, B) radiographie de thorax de face et de profil montrant le couteau en intra-thoracique; C, D) scanner thoracique montrant le couteau traversant l´axe aéro-digestif, un hémo-pneumothorax minime à droite et un emphysème sous-cutané

**Démarche diagnostique:** la radiographie du thorax de face et de profil (Figure 2 A, B) mettait en évidence le couteau qui traversait le thorax d´arrière en avant, de bas en haut et de gauche à droite, avec point d´entrée en regard de D5 et la pointe juste au-dessus de la carène. Il n´y avait pas de pneumo-médiastin ni d´élargissement du médiastin ni d´épanchement pleural. Le scanner thoracique (Figure 2 C, D) objectivait le corps étranger à porte d´entrée para-rachidienne droite à hauteur de D5 avec son bout distal en regard du TABC sans lésion de ce dernier. Il passe au-dessus du cœur, traverse l´œsophage et la trachée d´arrière en avant. Il y avait également des bulles d´air dans le médiastin, un hémo-pneumothorax droit de faible abondance et un emphysème sous-cutané en regard de la porte d´entrée. Le bilan pré-opératoire était sans particularité en dehors d´une hyperleucocytose à 16100/mm^3^.

**Figure 3:**
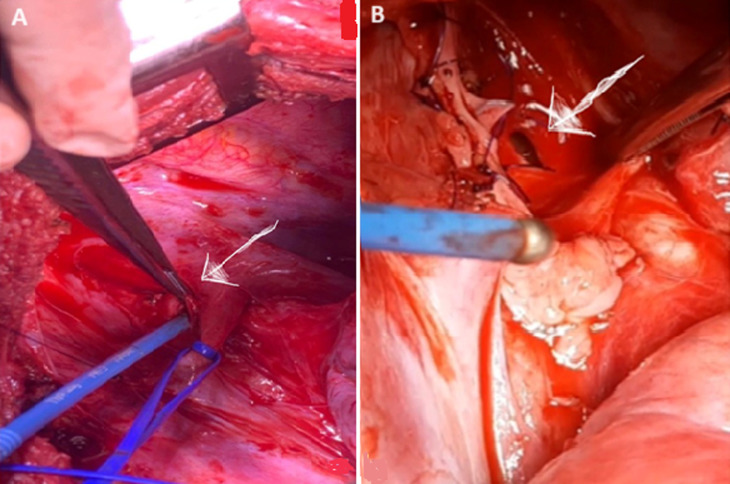
vue per-opératoire de la fistule oeso-trachéale; A) brèche œsophagienne sur son mur antérieur (flèche); B) brèche trachéale sur sa membraneuse postérieure (flèche)

**Traitement:** il a été admis au bloc opératoire à 14 heures du traumatisme. L´exploration chirurgicale par thoracotomie postéro-latérale droite avait permis de découvrir un couteau d´environ 15cm de long sur 4cm de large (Figure 1C), traversant le thorax d´arrière en avant avec son bout distal au niveau de la loge de Barety. Il a occasionné sur son trajet une plaie de la crosse de la veine azygos, une plaie pulmonaire lobaire supérieure droite d´environ 4 cm, une plaie œsophagienne transfixiante avec fistule oeso-trachéale d´environ 3 cm x 2 cm, juste au-dessus de la carène (entre le mur antérieur de l´œsophage et la membraneuse postérieure de la trachée (Figure 3).

Le corps étranger fut retiré. La fistule a été réparée par fermeture directe des 2 orifices trachéal et œsophagien par des points séparés au Monocryl 3/0: en 2 plans pour l´œsophage et en un plan pour la trachée. Un test au bleu de méthylène à travers une sonde naso-gastrique et un test au SSI avec insufflation d´air à travers la sonde trachéale d´intubation ont permis de vérifier l´étanchéité des sutures. Une plastie de recouvrement des sutures trachéales et bronchiques par de la plèvre médiastinale a été réalisée. Il a également bénéficié d´une ligature de la crosse de la veine azygos et une suture de la plaie parenchymateuse. Une toilette abondante de la cavité a été faite. Un drain thoracique en position médiastinale et un autre en basal de même qu´une sonde nasogastrique ont été placés. Une triple antibioprophylaxie à base de ceftriaxone, gentamycine et métronidazole a été instaurée pendant 10 jours. L´alimentation liquide puis semi-liquide a été débutée à J2 post-opératoire à travers la sonde naso-gastrique.

**Figure 4:**
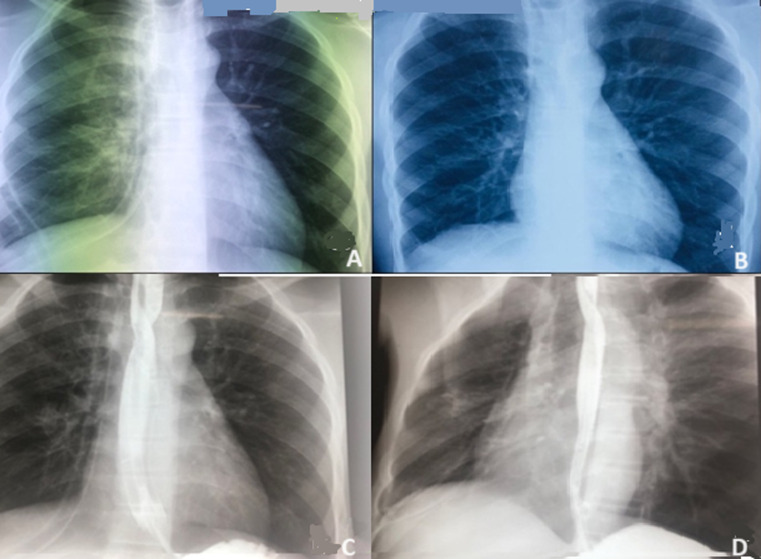
radiographies de contrôle post-opératoire: A) radiographie du thorax post-opératoire immédiat avec drains en place et petit pneumothorax droit apical; B) radiographie du thorax de contrôle à J45 post-opératoire; C, D) transit oeso-gastro-duodénal à la gastrografine de contrôle à J14 avec absence d´extravasation du produit de contraste

**Evolution:** les suites opératoires étaient simples avec une radiographie du thorax à J1 puis à J4 qui montrait un minime pneumothorax droit en apical avec un émoussement du cul de sac costo-diaphragmatique (Figure 4A); le transit œsogastroduodénal (TOGD) de contrôle fait à J14 ne retrouvait pas de fistule résiduelle (Figure 4B, C). L´ablation des drains et de la sonde naso-gastrique ont été faites à J14 et l´alimentation orale a été débutée sans incident. Il a été mis en exéat à J14 avec un suivi en consultation externe.

**Suivi:** au dernier contrôle à J45 post-opératoire, il n´avait pas de plainte, l´examen physique était normal avec une plaie opératoire cicatrisée et la radiographie du thorax était sans particularité (Figure 4D).

**Ressenti du patient:** le patient a apprécié la prise en charge rapide. Il s´est cependant posé beaucoup de questions par rapport à la période de jeûne. Les parents ont apprécié la prise en charge et l´évolution favorable après un suivi de 45 jours.

**Consentement éclairé:** le consentement éclairé du patient et de ses parents a été obtenu verbalement.

## Discussion

Les fistules oeso-trachéales acquises chez l´adulte sont rares. Elles surviennent le plus souvent dans un contexte de néoplasies malignes. Les fistules « bénignes » sont surtout secondaires à des pathologies infectieuses, traumatiques, caustiques, à des corps étrangers, des diverticules œsophagiens ou iatrogènes. Les étiologies traumatiques sont extrêmement rares. Elles constituaient 0,001% des lésions dans une étude de Reed [1] sur les traumatismes du thorax. Il peut s´agir alors d´un traumatisme fermé suite à un accident de la circulation chez un conducteur sans ceinture de sécurité, avec phénomène de compression et de décélération.

Le mécanisme serait une lacération partielle de la membraneuse postérieure de la trachée avec contusion du mur antérieur de l´œsophage suite à une compression de l´axe aéro-digestif entre le sternum et le rach, comme décrit par Chapman [2]. Un mécanisme direct, par traumatisme ouvert, comme dans notre cas, est très rare dans la littérature. Kanne [3] a décrit le cas d´une femme blessée par une balle d´une arme à feu. Le diagnostic est difficile et les symptômes peuvent apparaître immédiatement voire 38 ans plus tard [4]. Le maître symptôme est le signe d´Ono (toux lors de la déglutition). Il peut s´y associer une dyspnée, une dysphagie ou une odynophagie, une hémoptysie ou des hématémèses, une voix enrouée, un ballonnement abdominal, un emphysème sous-cutané cervico-thoracique [1]. Le diagnostic est fortement évoqué devant l´association d´un pneumothorax, d´un pneumo médiastin, d´un emphysème sous-cutané, d´un passage d´air à travers une sonde naso-gastrique lors de chaque inspiration.

Il est confirmé par un transit oeso-gastro-duodénal (montrant le passage du produit de contraste de l´œsophage vers l´arbre trachéo-bronchique) ou une tomodensitométrie (TDM) cervico-thoracique avec opacification digestive. L´endoscopie bronchique et digestive haute peuvent apporter des informations supplémentaires. Les symptômes frustes chez notre cas peuvent s´expliquer par le fait que le patient était à jeun jusqu´à l´intervention et cette dernière avait été réalisée dans un délai relativement court. La découverte a été fortuite en peropératoire après quelques signes évocateurs sur l´imagerie pré-opératoire (bulles d´air dans le médiastin, emphysème sous-cutané, pneumothorax, couteau traversant l´axe aéro-digestif). Le traitement des fistules oeso-trachéales est chirurgical. En effet, en l´absence de chirurgie, la mortalité est beaucoup plus élevée (80%) que chez les patients traités chirurgicalement (9,3%) [1]. Les tentatives de traitement non chirurgical sont décevantes et souvent réservées aux patients inopérables. Samalin [5] a obtenu de bons résultats après traitement par colle chimique (cyanoacrylate) chez 2 patients présentant des fistules oeso-bronchiques sur terrain de néoplasie maligne. Les endoprothèses sont difficiles à adapter au calibre exact des orifices fistuleux et posent le problème de leur possible migration (43% dans la série de Blackmon *et al*. [6].

Le traitement chirurgical dépend essentiellement du calibre, du siège et de l´ancienneté de la fistule. Pour les petites fistules oeso-trachéales relativement jeunes, la double suture directe réalisée chez notre patient est le traitement de choix, avec 2 plans de suture pour l´œsophage et un plan pour la trachée, complétée par une plastie de recouvrement par un lambeau de muscle intercostal, de péricarde ou de plèvre [7]. Les alternatives à cette technique en cas de fistule large ou ancienne sont nombreuses avec des résultats variables. La résection-suture trachéale segmentaire [8] (résection de la trachée sur quelques centimètres au niveau de la zone de fistule puis ré-anastomose termino-terminale par suture circulaire) nécessite une longueur de trachée suffisante pour une réparation termino-terminale. Duong et Beaulieux [9] ont proposé une bi-exclusion œsophagienne (sus et sous-fistuleux) avec rétablissement de la continuité digestive par un transplant gastrique ou colique, avec de bons résultats.

Les complications post-opératoires sont essentiellement d´ordres infectieux notamment dues au liquide salivaire resté en contact avec les sutures ou le liquide refluant de l´estomac, le risque de médiastinite et les infections pulmonaires. Ce risque est accru avec le délai de prise en charge. Stothert *et al*. [10] a montré les bons résultats des réparations précoces dans les 12 premières heures, contrastant avec les résultats médiocres survenant au-delà de ce délai. Pour ces derniers cas, une réparation différée, après préparation nutritionnelle et usage des techniques d´exclusion-reconstruction digestive est recommandée [9,10]. Une antibioprophylaxie à large spectre associée à une kinésithérapie respiratoire précoces permet également de réduire la morbidité post-opératoire [1]. Le risque de lâchage des sutures est également majoré par le mauvais état nutritionnel dû à la restriction alimentaire. C´est ainsi que les auteurs [10] recommandent une nutrition parentérale efficace et une alimentation par la sonde naso-gastrique dès 48h.

## Conclusion

Les fistules oeso-trachéales post-traumatiques par arme blanche sont extrêmement rares. Le diagnostic est difficile et il faut y penser devant une plaie thoracique postérieure transfixiante, passant par l´axe aéro-digestif, avec pneumothorax, pneumomédiastin et emphysème sous-cutané. La prise en charge chirurgicale précoce avec fermeture directe des orifices fistuleux et plastie de recouvrement des sutures trachéales et bronchiques nous a permis d´obtenir un bon résultat chez notre patient. Une bonne stratégie nutritionnelle et de prévention des infections est cependant nécessaire afin de réduire la morbi-mortalité péri-opératoire. Ces techniques simples et accessibles sont parfaitement reproductibles et adaptées dans nos pays en voie de développement.

## Remerciements

Les auteurs remercient tous ceux qui ont participé à l'élaboration de ce travail.
